# Vorschlag der rheumatologischen Fachgesellschaften im D-A-CH-Raum für eine einheitliche deutschsprachige Nomenklatur der Sjögren-Erkrankung

**DOI:** 10.1007/s00393-026-01798-1

**Published:** 2026-03-19

**Authors:** Diana Ernst, Thomas Dörner, Martin Aringer, Stephanie Finzel, Lisa Christ, Diana Dan, Alexandre Dumusc, Isabell Haase, Ina Kötter, Anna Meinecke, Ulf Müller-Ladner, Gabriela Riemekasten, Marc Schmalzing, Christof Specker, Christina Duftner, Martin Helmut Stradner, Paul Studenic, Anne-Kathrin Tausche, Jens Thiel, Ulf Wagner, Torsten Witte

**Affiliations:** 1 Medizinische Hochschule Hannover, Hannover, Deutschland; 2 Charité Universitätsmedizin Berlin Campus Charite Mitte: Charité Universitätsmedizin Berlin, Berlin, Deutschland; 3 Universitätsklinikum Carl Gustav Carus an der Technischen Universität Dresden, Dresden, Deutschland; 4 Universitätsklinikum Freiburg, Freiburg, Deutschland; 5 Universitätsklinik für Rheumatologie und Immunologie, Inselspital, Universitätsspital Bern, Universität Bern, Bern, Schweiz; 6 Service de rhumatologie, Centre Hospitalier Universitaire Vaudois, Lausanne, Schweiz; 7 Faculté de biologie et médecine, Université de Lausanne, Lausanne, Schweiz; 8 Universitätsklinikum Hamburg Eppendorf, III. Medizin, Sektion für Rheumatologie und Entzündliche Systemerkrankungen, Hamburg, Deutschland; 9 Justus-Liebig Universität Giessen, Abt. Rheumatologie und Klinische Immunologie, Campus Kerckhoff, Bad Nauheim, Deutschland; 10 Universitätsklinikum Schleswig-Holstein, Campus Lübeck, Lübeck, Deutschland; 11 Medizinische Klinik und Poliklinik 2, Universitätsklinik Würzburg, Würzburg, Deutschland; 12 Ev. Kliniken Essen-Mitte, Essen, Deutschland; 13 Univ.-Klinik für Innere Medizin II, Medizinische Universität Innsbruck, Innsbruck, Österreich; 14 Klinische Abteilung für Rheumatologie und Immunologie, Medizinische Universität Graz, Graz, Österreich; 15 Universitätsklinik für Innere Medizin III, Medizinische Universität Wien, Wien, Österreich; 16 Universitätsklinikum Leipzig, Leipzig, Deutschland; 17 Klinik für Rheumatologie und Immunologie, Medizinische Hochschule Hannover, Carl-Neuberg-Str. 1, 30625 Hannover, Deutschland; 18 Auenlandklinik Bad Bramstedt, Klinik für Rheumatologie und Immunologie, Bad Bramstedt, Deutschland; 19 Abteilung für Rheumatologie und Klinische Immunologie, Department Innere Medizin, Universitätsklinikum Freiburg, Freiburg, Deutschland

**Keywords:** Sjögren-Erkrankung, Sjögren-Syndrom, SjD, Primäres Sjögren-Syndrom, Konsensus, Sjögren’s disease, Sjögren’s syndrome, SjD, Primary Sjögren`s syndrome, Consensus

## Abstract

**Hintergrund:**

International wird das Sjögren-Syndrom jetzt als Sjögren’s disease (abgekürzt SjD) bezeichnet.

**Ziel der Arbeit:**

Es sollte ein Konsensus gefunden werden, wie das Sjögren-Syndrom künftig im deutschsprachigen Raum benannt wird.

**Material und Methoden:**

Von den drei Fachgesellschaften im D‑A-CH-Raum (Deutsche Gesellschaft für Rheumatologie und Immunologie e. V. [DGRh], Österreichische Gesellschaft für Rheumatologie und Rehabilitation [ÖGR], Schweizerische Gesellschaft für Rheumatologie [SGR]) wurden die Autorinnen und Autoren dieses Manuskripts benannt, um die internationalen Empfehlungen für den deutschsprachigen Raum umzusetzen.

**Ergebnisse:**

Der Begriff „Sjögren-Erkrankung“ sollte „Sjögren-Syndrom“ ersetzen. Die englische Abkürzung „SjD“ (Sjögren Disease) sollte auch auf Deutsch als Abkürzung für „Sjögren-Erkrankung“ verwendet werden. Der Begriff „assoziiert“ sollte anstelle von „sekundär“ verwendet werden für eine Sjögren-Erkrankung, die in Verbindung mit einer anderen klassifizierbaren systemischen Autoimmunerkrankung auftritt.

## Abstract (en)

**Background:**

Sjögren’s syndrome is now referred to internationally as Sjögren’s disease (abbreviated SjD).

**Objectives:**

The aim was to reach a consensus on how Sjögren’s syndrome should be referred to in German-speaking countries in the future.

**Material and methods:**

The authors of this manuscript were appointed by the three professional associations in the D‑A-CH region, the German Society for Rheumatology and Immunology (DGRh), the Austrian Society for Rheumatology (ÖGR) and the Swiss Society for Rheumatology (SGR) to implement the international recommendations for the German-speaking region.

**Results:**

The term “Sjögren-Erkrankung” should replace “Sjögren-Syndrom.” The English abbreviation “SjD” (Sjögren’s disease) should also be used in German as an abbreviation for “Sjögren-Erkrankung.” The term “associated” should be used instead of “secondary” for Sjögren’s disease that occurs in conjunction with another classifiable systemic autoimmune disease.

Aktuell wurde eine internationale Überarbeitung zur Nomenklatur des Sjögren-Syndroms, zukünftig Sjögren-Erkrankung, veröffentlicht, welches auf einer Zusammenarbeit von ACR, EULAR und Patientenorganisationen beruht [1]. Während Einigkeit bestand, den Namen Sjögren beizubehalten, gab es insbesondere von Seiten der Patientenvertreterinnen und -vertreter den dringenden Wunsch, die offizielle Nomenklatur von Sjögren-Syndrom in Sjögren-Erkrankung umzubenennen.

In einem substanziellen Einigungsprozess mit zwei Vorschlägen aus den USA und Europa und mehreren Delphi-Verfahren wurde unter Teilnahme von 79 Fachspezialistinnen und -spezialisten aus 28 Ländern und gemeinsam mit Patientenvertreterinnen und -vertretern beschlossen, „Sjögren(’s) syndrome“ in „Sjögren disease“, kurz SjD, umzubenennen [1].

Für den neuen Namen stimmten 77 % der Stimmberechtigten, während ein alternativer Vorschlag, die Umbenennung zu dem Akronym *S.J.Ö.G.R.E.N.’ (salivary gland swelling, joint pain/swelling, ocular/oral dryness, general symptoms, renal/respiratory manifestations, exocrinopathy, non-Hodgkin lymphoma),* nur ein Viertel der Stimmen erhielt. Dabei bleibt zu erwähnen, dass die Zustimmung für „Erkrankung“ bei den Patientinnen und Patienten noch deutlicher war als in der Ärzteschaft.

Die Fachgesellschaften im D‑A-CH-Raum (Deutsche Gesellschaft für Rheumatologie und Klinische Immunologie e. V. [DGRh], Österreichische Gesellschaft für Rheumatologie und Rehabilitation [ÖGR], Schweizerische Gesellschaft für Rheumatologie [SGR]) wollten sich dieser neuen Namensgebung anschließen und zukünftig von der Sjögren-Erkrankung sprechen und nominierten die Autorinnen und Autoren dieses deutschsprachigen Konsensus-Manuskripts für die Übersetzung und Überprüfung der internationalen Empfehlungen. Aus Gründen der Klarheit soll die englische Abkürzung SjD für die Sjögren-Erkrankung verwendet werden.

Das zweite Ergebnis des internationalen Konsensusprozesses war, dass die bislang oft nicht eindeutige Einteilung in primäre und sekundäre Sjögren-Erkrankung nicht mehr benutzt werden soll. Vielmehr soll von einer SjD mit oder ohne assoziierte autoimmune Erkrankungen gesprochen werden und damit „sekundär“ durch „assoziiert“ ersetzt werden.

Auch hier möchten sich die drei Fachgesellschaften dem Konsens anschließen und statt von einer „sekundären“ Sjögren-Erkrankung künftig zum Beispiel von einer „mit einer systemischen Sklerose [2] assoziierten Sjögren-Erkrankung“ sprechen. Eine Koexistenz von anderen autoimmunen, zum Teil organspezifischen Autoimmunerkrankungen ist sehr häufig [3]. Um den unterschiedlichen Phänotypen gerecht zu werden, soll dies nun insbesondere bei wissenschaftlichen Arbeiten berücksichtigt werden, und es soll von Sjögren-Patientinnen/Patienten mit und ohne assoziierte Autoimmunerkrankungen gesprochen werden. Unverändert bleibt, dass bei Erfüllung der jeweiligen Klassifikationskriterien auch der Begriff „Overlap-Syndrom aus …“ weiter verwendet werden kann, da im Prinzip alle rheumatischen Erkrankungen mit einer Sjögren-Erkrankung zusammen auftreten können und häufig die Sjögren-Erkrankung nach der bisher führenden Erkrankung auftritt.

Nach Ansicht der drei Fachgesellschaften und der Autorinnen und Autoren ist auch diese neue Nomenklatur für die Differenzierung der Sjögren-Erkrankung ein Schritt in die richtige Richtung, um Patientenkohorten besser vergleichen zu können. Im klinischen Alltag wird empfohlen, SjD als Abkürzung zu verwenden und nur optional primär oder (z. B. mit SSc) assoziiert zu ergänzen.

Zusammenfassend werden in Einklang mit der Task Force of Nomenclature of SjD (REF PMID: 40494962) folgende Änderungen vorgeschlagen:

1. Der Begriff „Sjögren-Erkrankung“ sollte „Sjögren-Syndrom“ ersetzen.
2. Die englische Abkürzung „SjD“ („Sjögren’s disease“) sollte auch auf Deutsch als Abkürzung für „Sjögren-Erkrankung“ verwendet werden.
3. Der Begriff „assoziiert“ sollte anstelle von „sekundär“ verwendet werden für eine Sjögren-Erkrankung, die in Verbindung mit einer anderen klassifizierbaren systemischen Autoimmunerkrankung auftritt.

## Fazit

Die Umbenennung vom Sjögren-Syndrom zur Sjögren-Erkrankung war das Ergebnis eines internationalen Konsensusprozesses zur eindeutigen Beschreibung und erfüllt einen dringenden Patientenwunsch. Gerade wegen des eindeutigen Patientenvotums befürworten die drei Fachgesellschaften aus dem D‑A-CH-Raum und die Autorinnen und Autoren die Umsetzung des Konsensus von EULAR und ACR im deutschen Sprachraum. Auch die Umbenennung von sekundärer Sjögren-Erkrankung zu assoziierter Sjögren-Erkrankung sollte mitgetragen werden. In jedem Fall wird dadurch sichergestellt, dass koexistente autoimmune Erkrankungen in wissenschaftlichen Arbeiten und Studien eine differenzierte Beachtung finden. Das sollte helfen, Antworten auf die Frage zu finden, inwieweit eine assoziierte SjD eine homogene Entität ist oder je nach Grunderkrankung spezifische Besonderheiten aufweist.

## References

[1] Ramos-Casals M, Baer AN, Brito-Zerón MdP et al (2025) 2023 International Rome consensus for the nomenclature of Sjögren disease. Nat Rev Rheumatol 21:426–437. 10.1038/s41584-025-01268-z 40494962

[2] Aringer M, Müller-Ladner U, Burkhardt H, Distler JH, Distler O, Graninger WB, Günther C, Hunzelmann N, Kiener H, Sticherling M, Sunderkötter C, Walker UA, Riemekasten G (2015) Gemeinsame deutschsprachige Nomenklatur für die systemische Sklerose. Z Rheumatol 74(2):100–103. 10.1007/s00393-014-1550-5 25805510

[3] Murray A, Gaedicke L, Zehrfeld N, MV A, T F, Benz S, Witte T, Skripuletz T, Seeliger T, Ernst D (2025) Sjögren’s disease is often associated with other autoimmune diseases. In: Deutsche Gesellschaft für Rheumatologie, Deutsche Gesellschaft für Orthopädische Rheumatologie (eds) Deutscher Rheumatologiekongress 2025. 53. Kongress der Deutschen Gesellschaft für Rheumatologie (DGRh), 39. Jahrestagung der Deutschen Gesellschaft für Orthopädische Rheumatologie (DGORh). Wiesbaden, 17.-20.09.2025, vol DocVK.62. German Medical Science GMS Publishing House, Düsseldorf

