# Erratum zu: Vorschlag der rheumatologischen Fachgesellschaften im D-A-CH-Raum für eine einheitliche deutschsprachige Nomenklatur der Sjögren-Erkrankung

**DOI:** 10.1007/s00393-026-01816-2

**Published:** 2026-04-27

**Authors:** Diana Ernst, Thomas Dörner, Martin Aringer, Stephanie Finzel, Lisa Christ, Diana Dan, Alexandre Dumusc, Isabell Haase, Ina Kötter, Anna Meinecke, Ulf Müller-Ladner, Gabriela Riemekasten, Marc Schmalzing, Christof Specker, Christina Duftner, Martin Helmut Stradner, Paul Studenic, Anne-Kathrin Tausche, Jens Thiel, Ulf Wagner, Torsten Witte

**Affiliations:** 1 Medizinische Hochschule Hannover, Hannover, Deutschland; 2 Charité Universitätsmedizin Berlin Campus Charite Mitte: Charité Universitätsmedizin Berlin, Berlin, Deutschland; 3 Universitätsklinikum Carl Gustav Carus an der Technischen Universität Dresden, Dresden, Deutschland; 4 Universitätsklinikum Freiburg, Freiburg, Deutschland; 5 Universitätsklinik für Rheumatologie und Immunologie, Inselspital, Universitätsspital Bern, Universität Bern, Bern, Schweiz; 6 Service de rhumatologie, Centre Hospitalier Universitaire Vaudois, Lausanne, Schweiz; 7 Faculté de biologie et médecine, Université de Lausanne, Lausanne, Schweiz; 8 Universitätsklinikum Hamburg Eppendorf, III. Medizin, Sektion für Rheumatologie und Entzündliche Systemerkrankungen, Hamburg, Deutschland; 9 Justus-Liebig Universität Giessen, Abt. Rheumatologie und Klinische Immunologie, Campus Kerckhoff, Bad Nauheim, Deutschland; 10 Universitätsklinikum Schleswig-Holstein, Campus Lübeck, Lübeck, Deutschland; 11 Medizinische Klinik und Poliklinik 2, Universitätsklinik Würzburg, Würzburg, Deutschland; 12 Ev. Kliniken Essen-Mitte, Essen, Deutschland; 13 Univ.-Klinik für Innere Medizin II, Medizinische Universität Innsbruck, Innsbruck, Österreich; 14 Klinische Abteilung für Rheumatologie und Immunologie, Medizinische Universität Graz, Graz, Österreich; 15 Universitätsklinik für Innere Medizin III, Medizinische Universität Wien, Wien, Österreich; 16 Universitätsklinikum Leipzig, Leipzig, Deutschland; 17 Klinik für Rheumatologie und Immunologie, Medizinische Hochschule Hannover, Carl-Neuberg-Str. 1, 30625 Hannover, Deutschland; 18 Auenlandklinik Bad Bramstedt, Klinik für Rheumatologie und Immunologie, Bad Bramstedt, Deutschland; 19 Abteilung für Rheumatologie und Klinische Immunologie, Department Innere Medizin, Universitätsklinikum Freiburg, Freiburg, Deutschland

**Erratum zu:**

**Z Rheumatol 2026**

https://doi.org/10.1007/s00393-026-01798-1

In der Online-First-Publikation wurde bei den Autoren Diana Ernst, Thomas Dörner, Martin Aringer, Stephanie Finzel, Isabell Haase, Ina Kötter, Anna Meinecke, Ulf Müller-Ladner, Gabriela Riemekasten, Marc Schmalzing, Christof Specker, Anne-Kathrin Tausche, Jens Thiel, Ulf Wagner und Torsten Witte angegeben, dass Sie für die Deutsche Gesellschaft für Rheumatologie und klinische Immunologe e. V. (DGRh) schreiben.

Korrekt ist jedoch: Deutsche Gesellschaft für Rheumatologie und klinische Immunologie e. V. (DGRh).

Bitte beachten Sie die korrekte Schreibweise.

Außerdem wurde bei Diana Dan und Alexandre Dumusc eine fehlerhafte Affiliation angegeben:

- Service du Rhumatologie, CHUV Hopital orthopedique, Lausanne, Schweiz

Bitte beachten Sie die korrekten Affiliations:

- Service de rhumatologie, Centre Hospitalier Universitaire Vaudois, Lausanne, Schweiz
- Faculté de biologie et médecine, Université de Lausanne, Lausanne, Schweiz

Wir bitten um Verständnis.

Die Autoren

